# Polystyrene nanoplastics and microplastics can act as Trojan horse carriers of benzo(*a*)pyrene to mussel hemocytes in vitro

**DOI:** 10.1038/s41598-021-01938-4

**Published:** 2021-11-17

**Authors:** Alberto Katsumiti, María Paula Losada-Carrillo, Marta Barros, Miren P. Cajaraville

**Affiliations:** 1 CBET Research Group, Department Zoology and Animal Cell Biology, Faculty of Science and Technology and Research Centre for Experimental Marine Biology and Biotechnology PiE, University of the Basque Country UPV/EHU, Leioa, Basque Country Spain; 2 Present Address: GAIKER Technology Centre, Basque Research and Technology Alliance (BRTA), Zamudio, Spain

**Keywords:** Animal biotechnology, Assay systems, Biologics, Environmental biotechnology, Cellular imaging, Membrane trafficking, Environmental sciences, Ocean sciences

## Abstract

In this work we studied the ability of polystyrene (PS) nanoplastics (NPs) and microplastics (MPs) to transfer benzo(a)pyrene (BaP) to mussel hemocytes and to produce toxic effects in vitro. For this, intracellular fate and toxicity of PS NPs (0.05 μm) and MPs (0.5 and 4.5 μm) alone or with BaP and of BaP alone were assessed. Particles of 0.05 and 0.5 µm largely aggregated in the exposure medium whereas presence of BaP reduced particle aggregation. Cells internalized PS NPs and MPs alone or with BaP and these were found inside and outside lysosomes, depending on their size. PS particles alone or with BaP were cytotoxic to hemocytes only at the highest concentrations tested. The same was true for most sublethal endpoints except for increased phagocytic activity provoked by NPs and 0.5 μm MPs at lower concentrations. Plastic particles appeared to be the main drivers for reduced plasma membrane integrity and increased phagocytic and lysosomal activities whereas BaP appeared to contribute more to reduced cell viability and phagocytosis and increased ROS production and genotoxicity. Overall, PS NPs and MPs can act as carriers of BaP to mussel hemocytes, rising concerns about risks plastics associated to pollutants may pose to aquatic organisms.

## Introduction

Nowadays, approximately 300 million tons of plastic are produced worldwide every year and more than 8 million tons of plastic end up in the ocean^1^. Due to its durability, it is estimated that all plastic ever produced still remains in the environment^2^.

In the marine environment, the most abundant plastic polymers are polystyrene (PS), polyethylene (PE), polypropylene (PP), the group of polyesters, polyamide and acrylics (PP&A) and polyvinyl chloride (PVC)^2,3^. These plastics can be found in the marine environment as large plastic pieces and as small fragments such as microplastics (MPs, < 5 mm) or nanoplastics (NPs, < 100 nm)^4,5^.

In the last years, NP and MP pollution has received special attention since they can easily accumulate and are difficult to remove from the environment and because they are more bioavailable to marine organisms than larger plastics^2,6^. Both NPs and MPs can be found in large quantities depending on the area. For instance, in heavily populated coastal areas in China, MP concentration is estimated to vary from 545 to 4137.3 particles/m^3^^7^. In other populated areas in Japan, Europe and North America, MP concentration is estimated to be lower (up to 1.5 particles/m^3^ in northwest Europe)^7^. In the northeast Atlantic Ocean (Bay of Biscay), MP concentration varies from 0.00098 to 0.35 particles/m^3^ ^3^. Nevertheless, due to lack of standardized methodology for sampling, extraction and analysis of MPs in environmental samples, it is difficult to determine actual exposure concentrations and levels of MPs accumulated in biota, but recent studies showed that the size fraction below 150 µm in size is being overlooked in most published studies relying on FTIR for polymer identification^8^.

Due to their small size, MPs can be easily confounded as food particles by many marine organisms, such as zooplankton, fish, mammals and specially by filter-feeder organisms such as bivalves^9–12^. In marine bivalves, in vivo studies have reported that exposure to NPs and MPs may cause harmful effects. Decrease in feeding and metabolic rates and in energy reserves have been reported in mussels exposed to NPs and MPs^11,13^. Adverse effects on growth, development and reproduction have also been associated with the exposure to NPs and MPs^14^. At cellular and subcellular levels, in vitro studies in hemocytes have suggested that NPs and MPs are internalized into the cells via endocytic and non-endocytic pathways, finally accumulating inside lysosomes^15–17^. Once internalized into the cells, NPs and MPs may cause cytotoxicity, oxidative stress, changes in enzyme activities, lysosomal membrane destabilization, immunotoxicity, genotoxicity and proteomic/transcriptomic changes^13–24^.

Further, due to their high surface to volume ratio and hydrophobicity, NPs and MPs can adsorb toxic chemicals from the surrounding seawater and transfer them to marine organisms, a phenomenon called “Trojan horse effect”^25,26^. Several persistent organic pollutants (POPs) such as polycyclic aromatic hydrocarbons (PAHs) have been detected in plastic particles collected from different beaches around the world^27,28^.

PAHs have been reported to be the most abundant class of POPs adsorbed to plastic particles^28,29^. Studies have reported PAH concentrations ranging from 1454 to 6002 ng/g in micro-sized pellets collected in the southern coast of Brazil^30^ and from 35.1 to 8725.8 ng/g in MPs collected from beaches in the Canary Islands^28^. In other coastal areas and open seas close to China, Japan and the Caribbean Sea, PAH concentrations in MPs ranged from 1 to 9300 ng/g ^27^. In MPs collected in the North Pacific Ocean Gyre, PAH concentrations in MPs ranged from 4 to 846 ng/g ^29^. These MPs may then work as accumulators of large amounts of PAHs, serving as vectors of these toxic compounds to marine organisms.

Different experimental studies in vivo have demonstrated the ability of NPs and MPs to transfer PAHs to marine bivalves^19,22,31–34^. These studies have reported bioaccumulation of PAHs such as pyrene^31^, fluoranthene^19,33^ and benzo(a)pyrene (BaP)^22,32^ in different tissues of bivalves, as well as alterations on different immunological and antioxidant parameters, neurotoxicity and genotoxicity. NPs and MPs may accumulate and transfer different amounts of PAHs to organisms depending on the size of the particles. González-Soto et al.^22^ reported that smaller PS MPs (0.5 μm) posed an increased hazard in terms of the transfer of BaP to mussel tissues than larger PS MPs (4.5 μm). Similarly, Tang et al.^23^ reported that toxicity of BaP was generally aggravated when associated with smaller MPs (500 nm) and mitigated when associated with larger ones (30 µm). Studies have also indicated that additive, synergistic or antagonistic effects of the combination of NPs or MPs and PAHs may occur. In mussels, Avio et al.^31^ reported higher genotoxicity (micronuclei frequency) of PS MPs with pyrene compared to MPs alone. Authors also reported specific modulations in transcriptional responses upon exposure to PS MPs compared to PS MPs with pyrene^31^. Similarly, González-Soto et al.^22^ reported that PS MPs with BaP induced higher levels of DNA strand breaks compared to PS MPs alone in mussels after 7 days exposure. Despite the relatively large amount of papers published in the last years on the Trojan horse ability of NPs and MPs to transfer PAHs to marine organisms, fundamental questions still remain concerning the intracellular fate and mode of action of NPs and MPs alone or in combination with PAHs.

In this work we aimed to assess the ability of PS NPs (0.05 μm) and MPs (0.5 and 4.5 μm) to transfer BaP to mussel *Mytilus galloprovincialis* hemocytes and to induce toxic effects in vitro*.* For this purpose, intracellular fate was studied by transmission electron microscopy (TEM) and confocal microscopy. Toxic effects of PS NPs and MPs alone or in combination with BaP were determined using in vitro assays for cell viability, plasma membrane integrity, phagocytic and lysosomal activities, intracellular ROS production and genotoxicity. Additionally, the behaviour of NPs and MPs alone or in combination with BaP was characterized in different media.

## Results

### Characterization of NPs and MPs alone or with BaP in different media

Dynamic Light Scattering (DLS) was used to assess hydrodynamic size and surface charge of NPs and MPs alone or in combination with BaP suspended in supplemented Basal Medium Eagle (BME) and distilled water (DW). Results showed marked differences in the aggregation state of the particles in the two media (Table 1). Aggregation was more pronounced in BME than in DW and differences were higher in 0.05 µm NP suspensions (DW: 184.3 nm; BME: 7700 nm) followed by suspensions of 0.5 µm MPs (DW: 742.6 nm; BME: 2328 nm) and 4.5 µm MPs (DW: 5009 nm; BME: 6777 nm) (Table 1). Aggregation was higher in suspensions of particles alone than in preparations with BaP (0.05 µm NPs: 4889 nm, 0.5 µm MPs: 873.6 nm and 4.5 µm MPs: 5584 nm in BME), indicating that presence of BaP may increase the stability of NP and MP suspensions (Table 1).

**Table 1:** Hydrodynamic size (Z-average) and surface charge (Z-potential) of NPs and MPs alone or in combination with BaP suspended in supplemented Basal Medium Eagle (BME) and in distilled water (DW).

	Media	Z-average (nm)	Z-potential (mV)
0.05 µm NPs	BME	7700 ± 283	− 22.6 ± 2.17
	DW	184.3 ± 82	− 10.2 ± 0.06
0.05 µm NPs + BaP	BME	4889 ± 305	− 22.6 ± 2.6
0.5 µm MPs	BME	2328 ± 47	− 23.0 ± 2.36
	DW	742.6 ± 56	− 13.2 ± 0.43
0.5 µm MPs + BaP	BME	873.6 ± 35	− 24.5 ± 0.4
	DW	699.2 ± 47	− 10.1 ± 0.25
4.5 µm MPs	BME	6777 ± 357	− 22.5 ± 1.99
	DW	5009 ± 188	− 53.7 ± 0.61
4.5 µm MPs + BaP	BME	5584 ± 476	− 15.2 ± 0.49
	DW	5237 ± 75	− 50.1 ± 0.43

Data are given as mean ± SD values (n = 3).

Concerning surface charge, all particles showed negatively charged surface in both media, with Z-potential values varying from − 10.1 to − 53.7 mV (Table 1). MPs of 4.5 µm showed higher absolute Z-potential values when suspended in DW than in BME, indicating higher stability (Table 1). In the case of 0.05 µm NPs and 0.5 µm MPs, particles suspended in DW showed lower absolute Z-potential values compared with particles suspended in BME (Table 1). BaP did not influence particles surface charge (Table 1).

### Intracellular fate of NPs and MPs alone or with BaP

**Figure 1:**
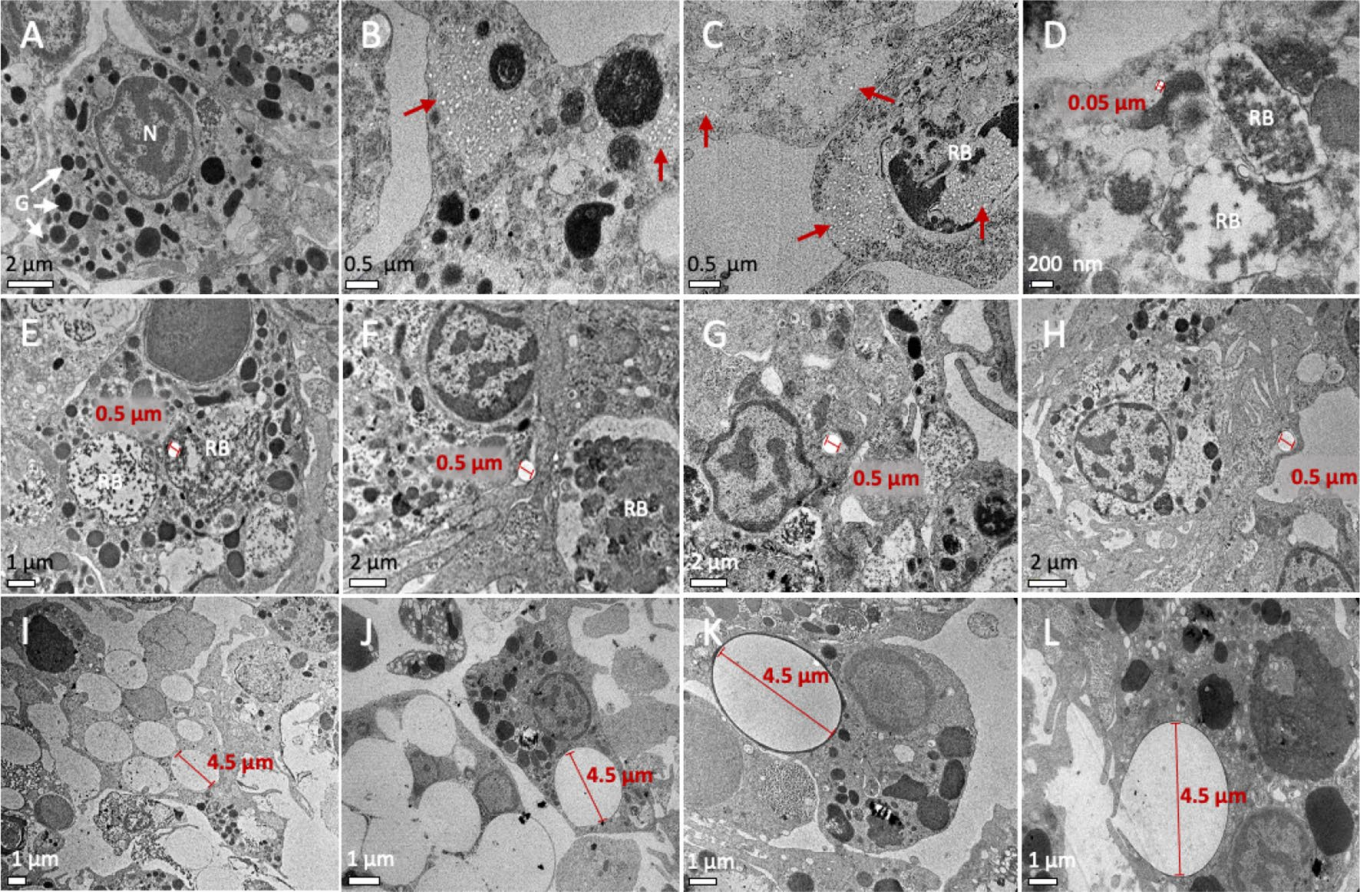
TEM images showing the intracellular localization of NPs and MPs in mussel hemocytes. (**A**) control cells, (**B**–**D**) hemocytes treated with 10^12^ part./mL of 0.05 μm NPs, (**E**–**H**) hemocytes treated with 10^9^ part./mL of 0.5 μm MPs and (**I**–**L**) hemocytes treated with 10^8^ part./mL of 4.5 μm MPs. Red arrows indicate the intracellular localization of NPs at low power magnification. Size of selected NPs and MPs is indicated (red lines). N, nucleus; G, granules; RB, residual bodies.

As seen in the transmission electron microscope (TEM) micrographs, cells treated with NPs and MPs showed signs of increased lysosomal and autophagic activities (Fig. 1). Compared to control cells (Fig. 1A), treated cells showed an increased number of large residual bodies, most containing lipofuscin-like pigment material (Fig. 1B–L). In cells treated with 0.05 μm NPs, particles were localized inside residual bodies and free in the cytosol (Fig. 1B–D). In cells treated with 0.5 μm MPs, particles were found outside the cells and inside residual bodies and other membrane-bound endo-lysosomal vesicles (Fig. 1E–G). MPs of 0.5 μm were also found surrounded and engulfed by cell surface extensions (Fig. 1H). In cells treated with 4.5 μm MPs, up to eight particles were found internalized per cell (Fig. 1I, J). MPs of 4.5 μm were engulfed through phagocytosis and found inside large membrane-bound phagosomal or phagolysosomal vesicles (Fig. 1K, L).

**Figure 2:**
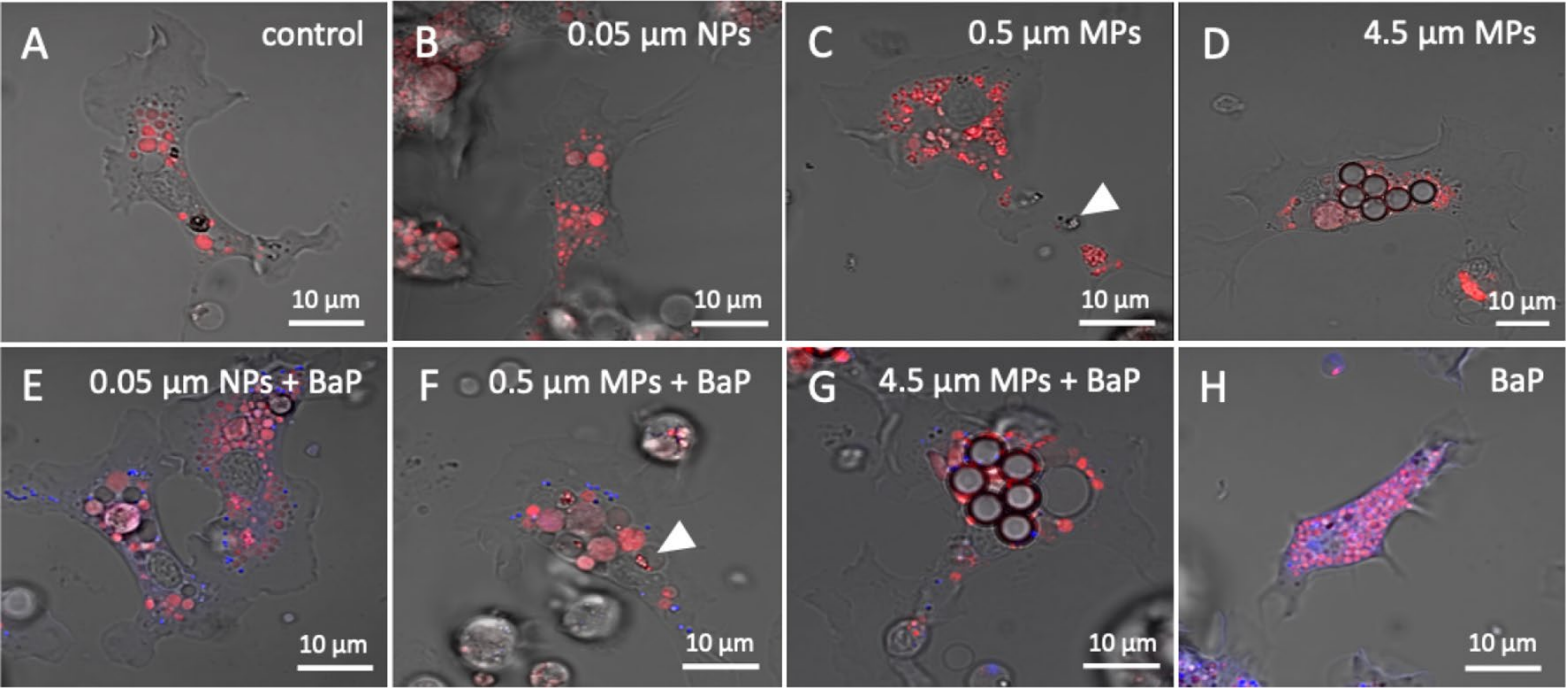
Confocal fluorescence microscope images showing lysosomes (red) and BaP (blue) in hemocytes exposed to 0.05 μm NPs (10^12^ part./mL) and to 0.5 and 4.5 μm MPs (10^8^ and 10^9^ part./mL, respectively) alone or in combination with BaP and to BaP alone. Arrowheads indicate structures resembling aggregates of 0.5 µm MPs.

In the confocal fluorescence microscope images, lysosomes labelled with LysoTracker™ were seen in red (Fig. 2 and supplementary Fig. S1) and BaP fluorescence in blue (Fig. 2E–H and supplementary Fig. S1). As expected, BaP was not detected neither in control cells nor in cells treated with particles alone (Fig. 2A–D and supplementary Figs. S1A–D online). In cells treated with 0.05 μm NPs alone, particles were not discernible (Fig. 2B and supplementary Fig. S1B). In cells treated with 0.5 μm MPs alone, structures resembling particle aggregates were found in the cytoplasm of the cells and apparently in lysosomes (Fig. 2C and supplementary Fig. S1C). In hemocytes exposed to 4.5 μm MPs alone, MPs (up to 6 particles/cell) were easily distinguished in cells cytoplasm surrounded by red fluorescence, thus indicating internalization into lysosomes (Fig. 2D and supplementary Fig. S1D). In cells treated with 0.05 μm NPs and 0.5 μm MPs with BaP, BaP fluorescence was found in discrete granules in the cytoplasm, some belonging to lysosomes (Fig. 2E, F and supplementary Fig. S1E, F). In cells exposed to 4.5 μm MPs with BaP, MPs were found surrounded by red (lysosomes) and blue (BaP) fluorescence (Fig. 2G and supplementary Fig. S1G). In cells treated with BaP alone (Fig. 2H and supplementary Fig. S1H), BaP fluorescence was found mainly internalized into lysosomes, as evidenced by the co-localization of blue and red fluorescence. Overall results show that NPs and MPs are able to carry BaP into the cells.

### In vitro toxicity

#### Cell viability

**Figure 3:**
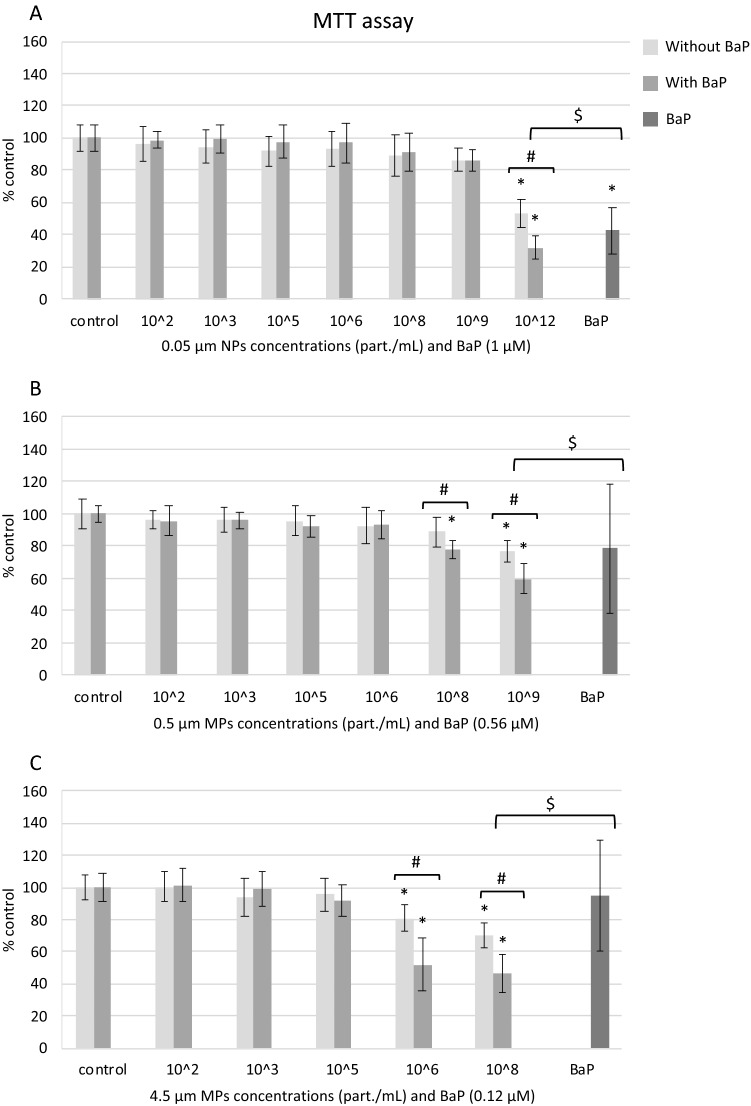
Cell viability (MTT assay) of mussel hemocytes exposed for 24 h to (**A**) 0.05 µm NPs, (**B**) 0.5 µm MPs and (**C**) 4.5 µm MPs alone or in combination with BaP and to BaP alone. Data are given as percentages with respect to control ± SD (n = 18). * indicates significant differences with respect to controls, # indicates significant differences between NPs/MPs with or without BaP and $ indicates significant differences between NPs/MPs with BaP and BaP alone.

NPs and MPs alone or in combination with BaP showed low cytotoxicity to mussel hemocytes, but treatments with BaP were more toxic than NPs or MPs alone (Fig. 3). Exposures to 0.05 µm NPs alone or with BaP decreased hemocytes viability only at the highest concentration tested (10^12^ part./mL) and cytotoxicity was significantly higher in the same treatment with BaP compared to NPs alone (Fig. 3A). In cells treated with 0.5 µm MPs alone, cell viability decreased only at the highest concentration tested (10^9^ part./mL), whereas in cells treated with 0.5 µm MPs with BaP, cell viability decreased at 10^8^ and 10^9^ part./mL with respect to controls (Fig. 3B). At both 10^8^ and 10^9^ part./mL, cytotoxicity was significantly higher in treatments with BaP compared to MPs alone (Fig. 3B). In hemocytes treated with 4.5 µm MPs alone or with BaP, cell viability decreased at 10^6^ and 10^8^ part./mL and cytotoxicity was significantly higher in treatments with BaP compared to MPs alone (Fig. 3C). BaP alone was cytotoxic to hemocytes only at the highest concentration tested (1 µM) compared to controls (Fig. 3A). At equivalent concentrations of BaP, the treatments NPs or MPs with BaP were significantly more toxic than BaP alone (Fig. 3).

#### Plasma membrane integrity

**Figure 4:**
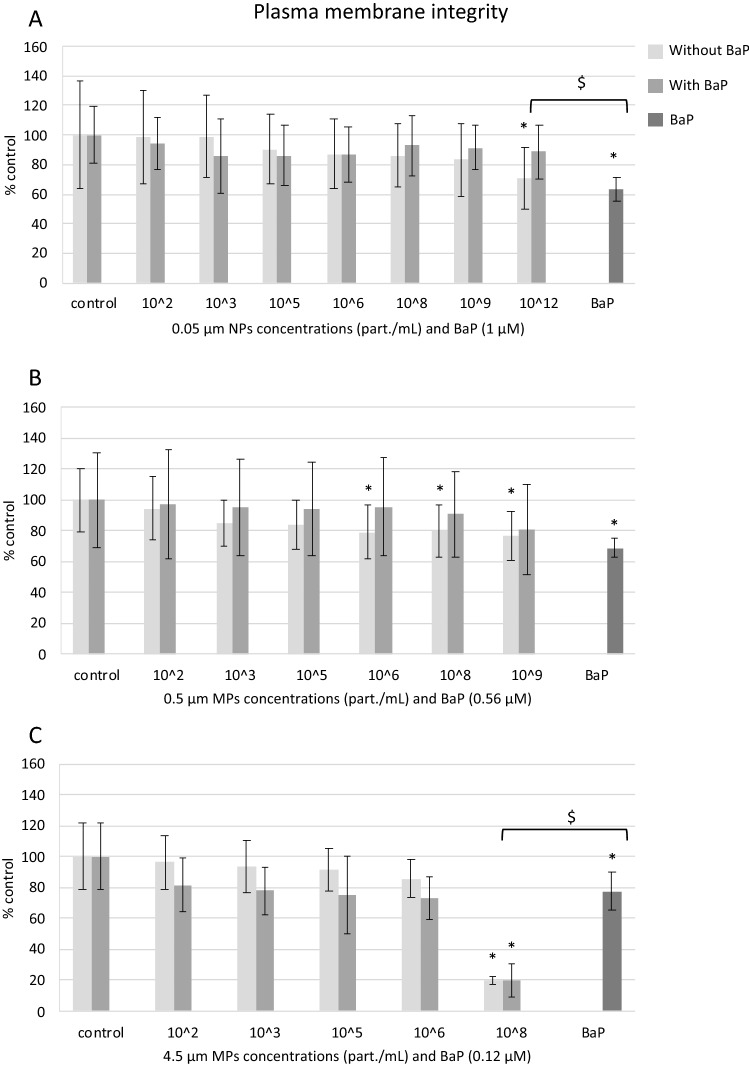
Plasma membrane integrity in mussel hemocytes exposed for 24 h to (**A**) 0.05 µm NPs, (**B**) 0.5 µm MPs and (**C**) 4.5 µm MPs alone or in combination with BaP and to BaP alone. Data are given as percentages with respect to control ± SD (n = 18). * indicates significant differences with respect to controls, $ indicates significant differences between NPs/MPs with BaP and BaP alone.

In exposures to NPs and MPs alone, plasma membrane integrity decreased significantly at 10^12^ part./mL of 0.05 µm NPs, 10^6^, 10^8^ and 10^9^ part./mL of 0.5 µm NPs and 10^8^ part./mL of 4.5 µm MPs with respect to controls (Fig. 4A–C). In cells treated with NPs and MPs with BaP, only 10^8^ part./mL of 4.5 µm MPs with BaP significantly disrupted plasma membrane integrity, at levels similar to 4.5 µm MPs alone at the same concentration (Fig. 4C). BaP alone reduced plasma membrane integrity at the three concentrations tested (Fig. 4A–C). At equivalent concentrations of BaP, BaP alone was more toxic than 0.05 µm NPs with BaP but less toxic than 4.5 µm MPs with BaP (Fig. 4A, C).

#### Phagocytic activity

**Figure 5:**
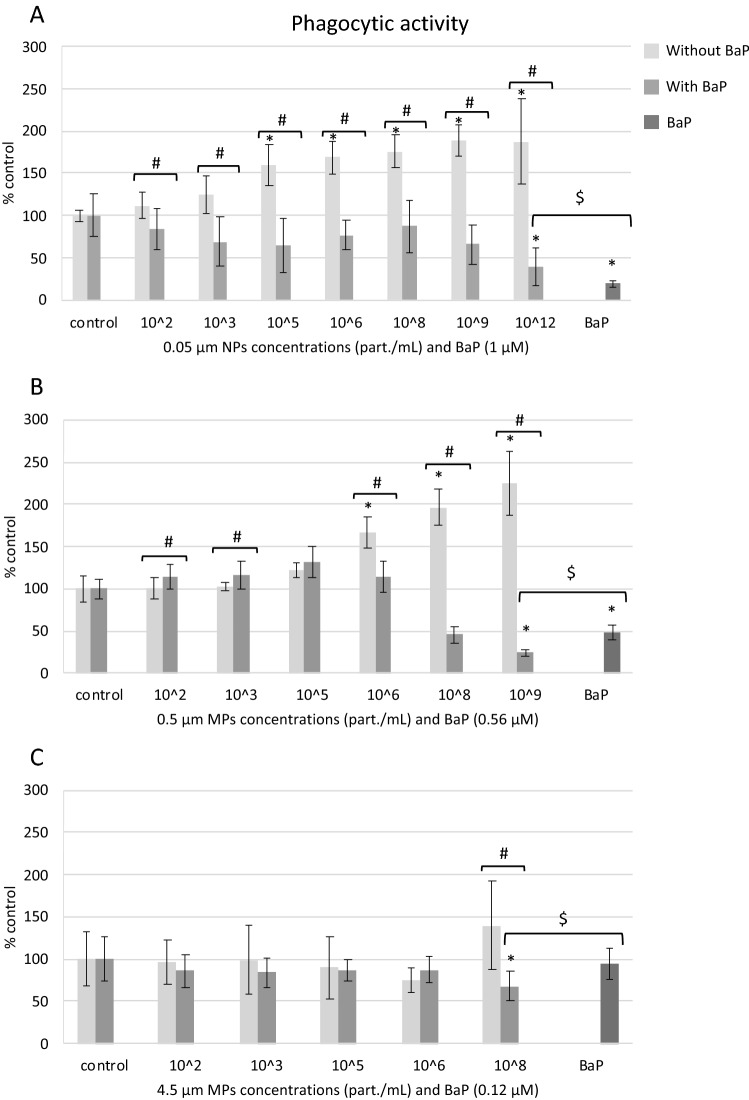
Phagocytic activity in mussel hemocytes exposed for 24 h to (**A**) 0.05 µm NPs, (**B**) 0.5 µm MPs and (**C**) 4.5 µm MPs alone or in combination with BaP and to BaP alone. Data are given as percentages with respect to control ± SD (n = 18). * indicates significant differences with respect to controls, # indicates significant differences between NPs/MPs with or without BaP and $ indicates significant differences between NPs/MPs with BaP and BaP alone.

Treatments with 0.05 μm NPs and 0.5 μm MPs alone increased hemocytes phagocytic activity compared to controls (Fig. 5A, B). Thus, phagocytic activity increased in hemocytes treated with 10^5^ to 10^12^ part./mL of 0.05 μm NPs and 10^6^ to 10^9^ part./mL of 0.5 μm MPs (Fig. 5A, B). In cells exposed to 10^8^ part./mL of 4.5 μm MPs alone, phagocytic activity was higher but not significantly different from that in control cells (Fig. 5C). Hemocytes phagocytic activity decreased in exposures to the highest concentrations of NPs and MPs with BaP (Fig. 5A–C). Phagocytic activity was also reduced in treatments to 0.56 and 1 μM of BaP alone (Fig. 5A, B). At equivalent concentrations of BaP, BaP alone inhibited phagocytosis more than NPs with BaP but less than MPs with BaP (Fig. 5A–C).

#### Lysosomal acid phosphatase (AcP) activity

**Figure 6:**
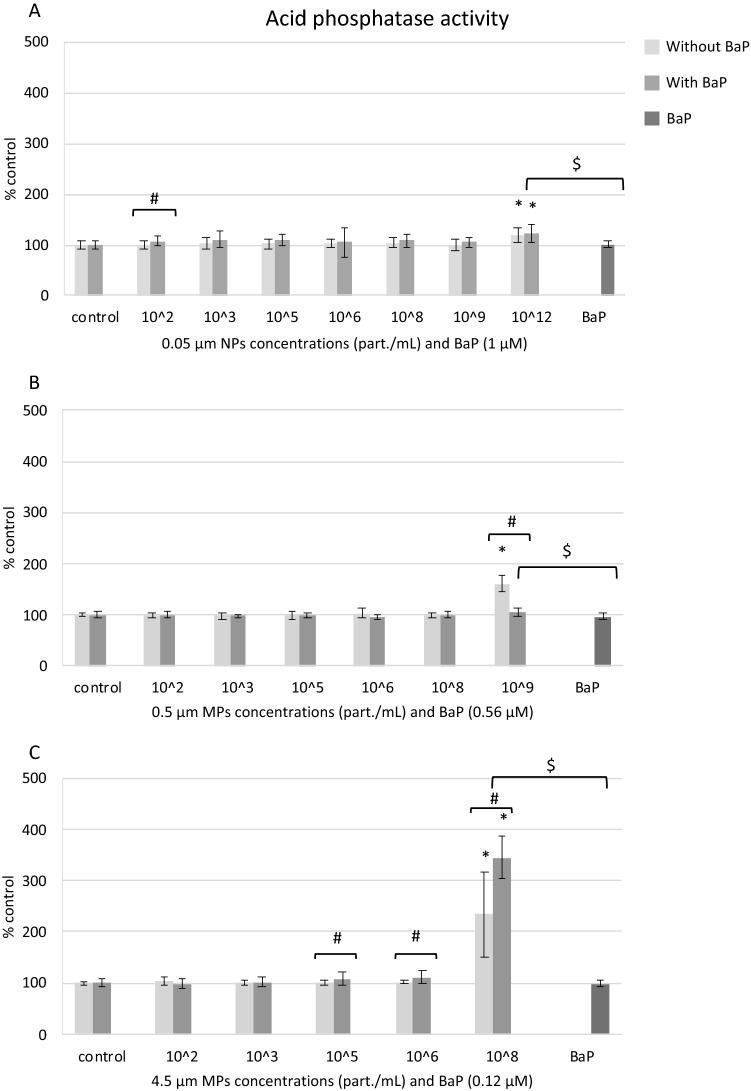
Lysosomal AcP activity in mussel hemocytes exposed for 24 h to (**A**) 0.05 µm NPs, (**B**) 0.5 µm MPs and (**C**) 4.5 µm MPs alone or in combination with BaP and to BaP alone. Data are given as percentages with respect to control ± SD (n = 18). * indicates significant differences with respect to controls, # indicates significant differences between NPs/MPs with or without BaP and $ indicates significant differences between NPs/MPs with BaP and BaP alone.

AcP activity increased in hemocytes exposed to the highest concentrations of NPs and MPs alone and in cells exposed to the highest concentrations of 0.05 μm NPs and 4.5 μm MPs with BaP (Fig. 6A–C). AcP activity was especially induced in exposures to 4.5 μm MPs at 10^8^ part./mL alone and with BaP (Fig. 6C). Comparing treatments with NPs and MPs alone or with BaP, AcP activity was significantly higher in exposure to 0.5 μm MPs alone compared to 0.5 μm MPs with BaP (Fig. 6B) but lower in hemocytes treated with 4.5 μm MPs alone compared to 4.5 μm MPs with BaP (Fig. 6C). BaP did not affect AcP activity at any concentration tested (Fig. 6). At equivalent concentrations of BaP, AcP activity was higher in hemocytes treated with NPs and MPs with BaP than in cells treated with BaP alone (Fig. 6A–C).

#### Reactive oxygen species (ROS) production

**Figure 7:**
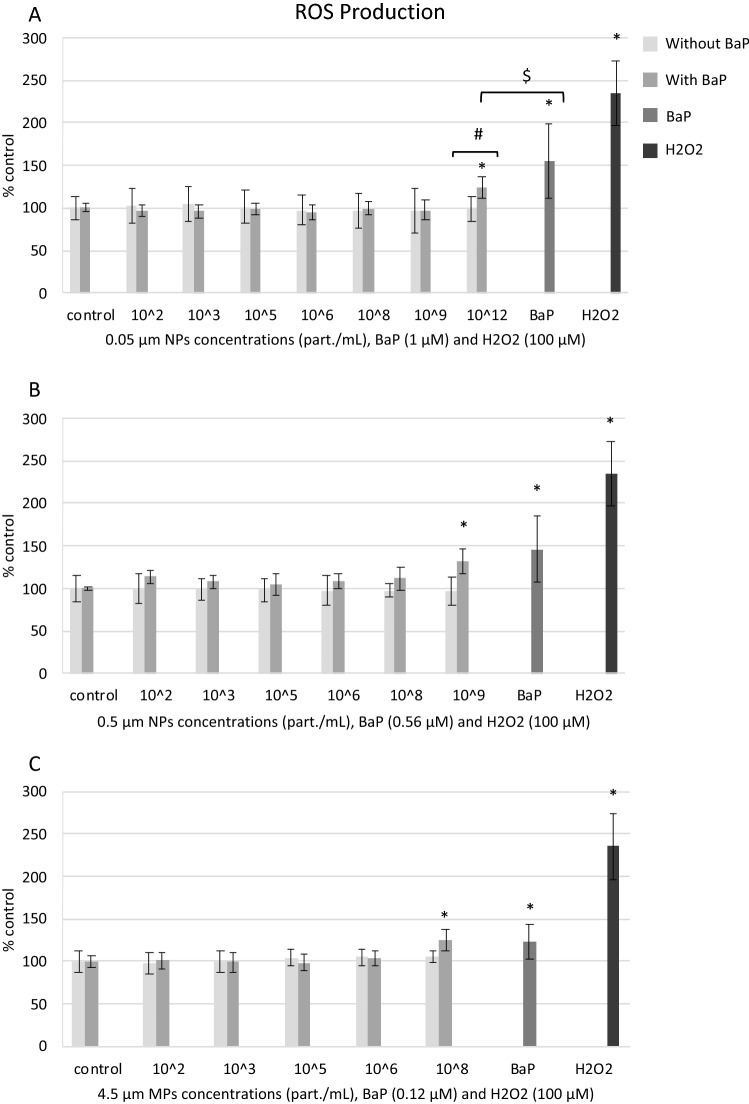
ROS production in mussel hemocytes exposed for 24 h to (**A**) 0.05 µm NPs, (**B**) 0.5 µm MPs and (**C**) 4.5 µm MPs alone or in combination with BaP, to BaP alone and to 100 µM H_2_O_2_ (positive control). Data are given as percentages with respect to control ± SD (n = 18). * indicates significant differences with respect to controls, # indicates significant differences between NPs/MPs with or without BaP and $ indicates significant differences between NPs/MPs with BaP and BaP alone.

Exposures to NPs or MPs alone did not induce ROS production in mussel hemocytes compared to controls, but when combined with BaP, NPs and MPs induced ROS production at the highest concentrations tested with respect to controls (Fig. 7A–C). BaP alone significantly increased ROS production at the three concentrations tested (Fig. 7A–C). At equivalent concentration of BaP, BaP alone induced a higher ROS production than 0.05 µm NPs with BaP (Fig. 7A).

#### Comet assay

**Figure 8:**
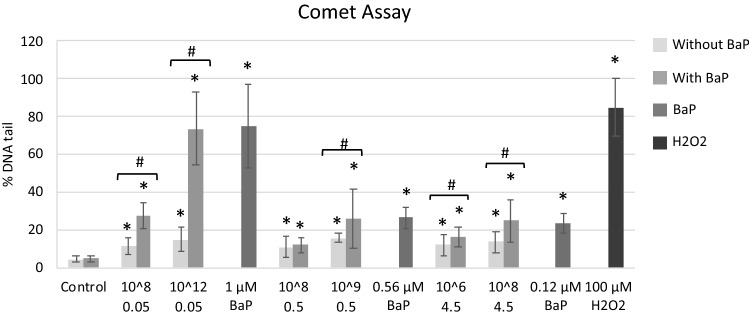
DNA damage in mussel hemocytes exposed for 24 h to 0.05 µm NPs, 0.5 µm MPs and 4.5 µm MPs alone or in combination with BaP, to BaP alone and to 100 µM H_2_O_2_ (positive control). Data are given as % DNA in tail ± SD (n = 100). * indicates significant differences with respect to controls, # indicates significant differences between NPs/MPs alone or with BaP.

NPs and MPs alone or with BaP produced significantly more DNA strand breaks at all concentrations tested compared to controls (Fig. 8). NPs and MPs with BaP were significantly more genotoxic than NPs and MPs alone (Fig. 8). BaP was genotoxic at the three concentrations tested (Fig. 8). At the highest concentration tested (1 μM), BaP was almost as genotoxic as the positive control (100 μM H_2_O_2_) (Fig. 8). At equivalent BaP concentrations, BaP alone induced similar levels of DNA damage as NPs and MPs with BaP (Fig. 8).

## Discussion

In this work we evaluated the ability of polystyrene (PS) NPs (0.05 μm) and MPs (0.5 μm and 4.5 μm) to transfer BaP to mussel hemocytes and to induce toxic effects in vitro. First, particles behaviour was characterized in different media and then, cells were exposed to a range of concentrations of NPs and MPs alone or with BaP in order to assess their intracellular fate and toxicity. Selected concentrations included those considered environmentally realistic^29^. Cells were also exposed to BaP at 1 μM, 0.56 μM and 0.12 μM, which correspond to the concentrations of BaP present in 10^12^ part./mL of 0.05 μm NPs with BaP and the concentrations of BaP adsorbed to 10^9^ part./mL of 0.5 μm MPs and to 10^8^ part./mL of 4.5 μm MPs with adsorbed BaP, respectively^35^.

Regarding particles behaviour in suspension, results showed that particle aggregation occurred depending on the size of the particles, the suspension media and the presence of BaP. When suspended in the BME cell culture medium, 0.05 µm NPs alone or with BaP aggregated largely reaching 4.8 to 7.7 µm, while MPs of 0.5 µm aggregated mostly in suspensions without BaP reaching up to 2 µm and MPs of 4.5 µm alone or with BaP did not aggregate. As already reported by other authors in different exposure media^16,17,36–38^, smaller PS particles are generally more prone to aggregation than larger ones. Song et al.^37^ reported that larger PS MPs-COOH (200 nm) have higher stability than smaller PS NPs-COOH (50 nm) in CaCl_2_ solution. Similarly, Dong et al.^36^ reported that smaller PS MPs (0.1–0.6 μm) aggregated more rapidly than larger ones (0.8–1.5 μm) in seawater. The use of physiologically relevant media such as filtered serum of mussel hemolymph does not prevent aggregation of NPs and MPs^16,17^ while it can add variability in exposure conditions compared to the use of a standard medium with known composition such as BME. In spite of DLS results indicating a substantial aggregation in BME, especially in case of NPs, individual particles of 0.05, 0.5 and 4.5 μm were identified by TEM within the cells exposed to the respective NPs or MPs. This appears to suggest that particles interacted with cells plasma membrane and became internalized individually, as discussed later.

Comparing particle stability in the two media, aggregation was lower in DW than in BME, possibly due to the higher ionic strength of the latter. As defined by the Derjaguin-Landau-Verwey-Overbeek (DLVO) theory, the colloidal stability of particles depends on the balance between the repulsive electrical double layer forces and the attractive van der Waals force. In salty media, van der Waals interaction dominates as the effective repulsion between NPs or MPs decreases by the increased concentration of electrolytes^39^. Thus, the high salt concentrations present in the BME increased the rate of aggregation of NPs and MPs resulting in decreased particles stability. On the other hand, aggregation was lower in particles with associated BaP. It has been reported that the presence of organic matter may increase particles stability^38^. Organic molecules bind to the particle surface via adsorption or hydrophobic interaction, increasing the steric and electrostatic repulsion between particles thus reducing aggregation^39^.

As to surface charge, NPs and MPs alone or with BaP showed a consistent negative surface charge, with Z potential values varying from − 10.1 to − 53.7 mV, in line with results in other exposure media^16,17^. MPs of 4.5 µm alone or with BaP showed higher absolute Z potential values in DW compared to suspensions in BME, thus exhibiting a higher stability in the former. Conversely, particles of 0.05 and 0.5 µm showed lower absolute Z potential values in DW compared to BME, indicating that, for small particles, the greater surface to volume ratio may favour the adsorption of medium components (eg. amino acids, vitamins) promoting a higher particle stability^40^.

**Figure 9:**
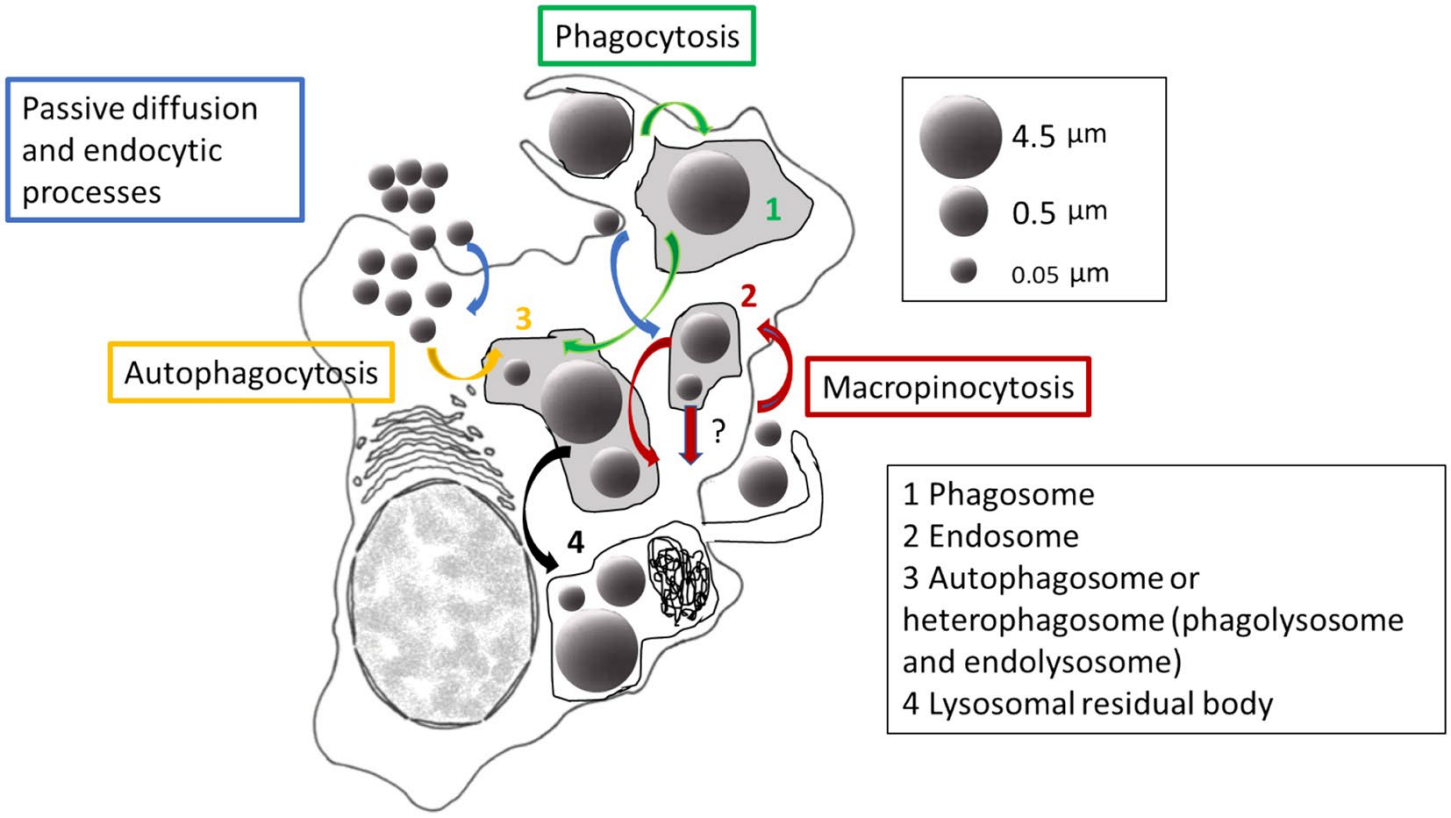
Simplified schematic diagram of a model for uptake and trafficking of PS NPs and MPs in mussel hemocytes. NPs formed large aggregates in the BME culture medium but were internalized and found in the cytosol and inside membrane-bound vesicles, indicating possible entrance by passive diffusion and endocytic processes, respectively. Also, it could be that NPs found in the cytosol escaped from the endo-lysosomal system after internalization (?). MPs of 0.5 μm were found extracellularly surrounded by cytoplasmic projections and intracellularly within membrane-bound vesicles suggesting internalization via macropinocytosis although other endocytic pathways could also be involved. NPs and MPs of 0.5 μm could reach the endosomes and endolysosomes. Finally, MPs of 4.5 μm appeared to enter in the hemocytes by phagocytosis and were found inside vesicles resembling phagosomes or phagolysosomes. Both by autophagic or heterophagic pathways, the final fate of NPs and MPs could be large tertiary lysosomes or residual bodies containing lipofuscins, which were more abundant than in control cells. It could be that these residual bodies were exocytosed from the cells at a later stage.

Particle properties such as surface charge, shape and size as well as the cell type are main determinants of uptake mechanisms and intracellular fate of NPs and MPs^41^. According to TEM analysis, NPs were found inside membrane-bound vesicles, especially within large tertiary lysosomes or residual bodies containing lipofuscins, which were more abundant than in control cells. This might indicate involvement of endocytic mechanisms and also of autophagy, as increased lipofuscin in mussel digestive cells is indicative of oxidative cell injury and autophagic dysfunction^42^. Nanoparticles have been suggested to either induce the process of autophagy or cause its dysfunction^43^. NPs were also found free in the cytosol, which is consistent with their internalization through diffusion across the plasma membrane, although it could also indicate that they escaped from the endo-lysosomal system after internalization^43^. MPs of 0.5 μm were found extracellularly surrounded by cytoplasmic projections and intracellularly within membrane-bound vesicles suggesting internalization via macropinocytosis although other endocytic pathways could also be involved^43^. Finally, MPs of 4.5 μm appeared to enter in the hemocytes by phagocytosis and were found inside vesicles resembling phagosomes or phagolysosomes^44^, in line with results of confocal microscopy. As in exposures to NPs, cells exposed to both sizes of MPs showed an increased amount of residual bodies and lipofuscins indicative of enhanced lysosomal and autophagic activity. Overall, as summarized in Fig. 9, our results agree well with studies indicating that NPs (0.05 μm or less) can be internalized by ATP-independent passive transport or endocytic mechanisms^41,43,45,46^, while small MPs (0.5 μm or less) can be internalized through clathrin or caveolae-mediated endocytic pathways or via macropinocytosis, and larger MPs (approximately 1 μm or larger) are taken up by phagocytosis^17,44,47–50^.

Confocal microscopy allowed to determine the intracellular fate of BaP in cells exposed to NPs with BaP and in cells exposed to MPs with adsorbed BaP, following Batel et al.^51^. Our results showed that PS NPs and MPs may act as Trojan horse carriers of BaP into mussel hemocytes. BaP fluorescence partly co-localized with labelled lysosomes, that could correspond to BaP associated to plastic particles incorporated by the different endocytic uptake routes mentioned before. BaP was also found outside of lysosomes, possibly due to entry of particles by diffusion or other non-endocytic mechanisms or by escaping the endo-lysosomal system after entry, as mentioned before. In exposures to BaP alone, BaP was found throughout the cell, mainly internalized into lysosomes, as shown before in mussel digestive cells upon exposure of mussels to BaP^42^. As far as we know, there are no previous studies on the intracellular localization of BaP or other PAHs associated to NPs or MPs. As to the localization of NPs or MPs alone, studies on human cells showed that PS NPs^52^ and PS MPs of 500 nm or less^53,54^ accumulated in the cytosol and inside lysosomes whereas larger PS particles (5 µm) accumulated mainly inside lysosomes^54^. In oyster hepatopancreas cells, Gaspar et al.^15^ reported that 50 nm PS NPs accumulated inside lysosomes and endosomes. In mussel hemocytes, PS particles of 50 nm, 100 nm and 1 μm were mostly co-localized with lysosomes except for 50 nm NPs that were also found in the cytoplasm outside lysosomes^17^. Our results of confocal microscopy are in accordance with these findings and also with TEM results.

Once inside the cells, both plastic particles and associated BaP could cause an array of toxic effects. Effects of NPs and MPs alone on cell viability (MTT assay) occurred only at relatively high concentrations^29^. Thus, cell viability decreased significantly upon exposures to 10^12^ part./mL (50 μg/mL) of 0.05 μm PS NPs; 10^9^ part./mL (50 μg/mL) of 0.5 μm PS MPs and 10^6^ part./mL (50 μg/mL) and 10^8^ part./mL (5000 μg/mL) of 4.5 μm PS MPs. In accordance with our results, other authors also reported low cytotoxicity of PS NPs and MPs in different cell models in vitro^21,54,55^. For the three sizes of particles tested cytotoxicity occurred at PS concentrations of 50 μg/mL or higher. Comparing the cytotoxicity of the three sizes of particles at the same PS mass concentration of 50 μg/mL, 0.05 μm NPs were more toxic (47.16% decrease of cell viability) than 0.5 μm and 4.5 μm MPs (23.35% and 29.67% decrease of cell viability, respectively). Results agree with the idea that smaller particles show higher bioreactivity due to their higher surface to volume ratio compared to larger particles^56^. Hwang et al.^57^ reported that smaller PP MPs (20 μm) were more toxic than larger ones (25–200 μm) to murine immune cells. Similarly, Stock et al.^55^ reported that smaller PS MPs (1 μm) exerted higher cytotoxicity than larger PS MPs (4 and 10 μm) to Caco-2 cells.

In cells treated with NPs or MPs with BaP, cytotoxicity was higher than in cells exposed to PS particles alone. BaP alone was cytotoxic to hemocytes only at the highest concentration tested (1 μM, the same concentration present in 10^12^ part./mL of 0.05 μm NPs). Previous studies in vitro have already reported the cytotoxicity of BaP in mussel hemocytes^58,59^. In mussels exposed in vivo to the same PS MPs used in this work (0.5 and 4.5 µm) alone or with BaP, González-Soto et al.^22^ reported larger effects on cell viability and catalase activity in hemocytes from mussels exposed to MPs with BaP compared to animals exposed to MPs alone. Similarly, other studies in vivo with mussels have also reported significant effects of MPs with PAHs in hemocytes^31,32^. In the human cell line A549 exposed in vitro to 190 nm PS MPs alone or with BaP and to BaP alone, PS MPs with BaP were more toxic than MPs or BaP alone^60^. Thus, the increased cytotoxicity of PS particles with BaP to mussel hemocytes appeared to be due to the presence of BaP carried into the cells, as demonstrated by confocal microscopy.

Similar to results on cell viability, plasma membrane integrity decreased significantly only at high concentrations of exposure to plastic particles, e.g., 10^12^ part./mL (50 μg/mL) of 0.05 µm NPs, 10^6^, 10^8^ and 10^9^ part./mL (0.5 to 50 μg/mL) of 0.5 µm MPs and 10^8^ part./mL (5000 μg/mL) of 4.5 μm MPs. Similarly, comparing the effects of the three sizes of particles at the same PS mass, smaller NPs and MPs exerted a higher impact on plasma membrane integrity than 4.5 μm MPs. Rossi et al.^61^ used molecular simulations to determine the effect of PS particles on model biological membranes and found that PS particles severely affected membrane organization and integrity especially at high concentrations. BaP alone significantly reduced plasma membrane integrity at the three concentrations tested. BaP is known to react with phospholipid membranes causing swelling and expansion, altering the structure and functionality of the membranes and compromising their integrity^62^. Comparing effects of plastic particles with associated BaP to effects of BaP alone at equivalent concentrations, no significant differences were seen for 0.5 µm MPs whereas plasma membrane integrity was reduced in BaP alone compared to NPs with BaP but increased in BaP alone compared to 4.5 μm MPs with adsorbed BaP. This might indicate a higher impart of larger 4.5 μm plastic particles on plasma membrane integrity compared to BaP effect.

Phagocytosis is the primary cellular defense reaction in invertebrate hemocytes and is first triggered by interaction of particles with cells plasma membrane. Mussel hemocytes, especially granulocytes, have long been known to actively phagocytose latex and zymosan particles in vitro^44^. Our results showed that exposure to PS NPs and small 0.5 µm MPs alone induced hemocytes phagocytosis of zymosan particles, an immunostimulatory effect that is widely reported for mussel hemocytes exposed to other nanoscale particles^63,64^. Importantly, for 0.5 μm MPs this effect occurred at the same concentrations with decreased plasma membrane integrity whereas for NPs the increase in zymosan phagocytosis occurred at concentrations that did not disturb plasma membrane integrity. On the other hand, hemocytes exposed to larger 4.5 μm MPs were not more active in zymosan phagocytosis than controls possibly because they were already loaded with MP particles, as seen by TEM. Studies on LDPE MPs reported an increase of hemocytes phagocytosis^32^, however other in vivo studies on PS and PE particles in the range 100–1000 μm ^31^ or in vitro studies on amino modified 50 nm PS NPs^18^ or fluorescent 1 μm MPs^16^ reported the opposite. Surface characteristics of NPs and MPs such as the presence of functional groups and surface charge and presence of a protein corona are decisive for particles recognition and internalization^11,18^. Further, methods to measure phagocytic activity differ largely; while some studies have measured phagocytosis of plastic particles themselves using flow cytometry (e.g.,^16^) we studied here phagocytosis of zymosan in cells exposed to NPs and MPs.

Conversely to findings with PS particles alone, in cells exposed to PS NPs and MPs with BaP and to BaP alone phagocytic activity was reduced. This immune inhibitory effect was significant with respect to controls at highest concentrations tested for the three sizes of plastics with BaP but differences between plastics alone versus plastics with BaP were already significant at 10^2^ part./mL of 0.05 µm NPs and 0.5 µm MPs. Reduced phagocytosis could be due to a shift in the recognition of the PS particles caused by the presence of BaP on the surface of particles. Since phagocytic activity requires an intact and functional actin cytoskeleton^65^, another possible explanation could be that BaP affected the integrity of the actin cytoskeleton of mussel hemocytes^58,59^ thus reducing phagocytosis of zymosan. Therefore, phagocytosis results suggest not only a carrier or Trojan horse effect of NPs and MPs but also a differential modulation of immune response in hemocytes caused by PS particles and BaP.

The uptake and trafficking of NPs and MPs at high concentrations along the endo-lysosomal system of hemocytes as well as the increased lysosomal and autophagic activities observed by TEM can be related to increased activities of lysosomal acid hydrolases. In agreement, AcP activity increased significantly in hemocytes exposed to the highest concentrations of 0.05 μm NPs (10^12^ part./mL or 50 μg/mL) alone or with BaP, 0.5 μm MPs (10^9^ part./mL or 50 μg/mL) alone, and 4.5 μm MPs (10^8^ part./mL, 5000 μg/mL) alone or with BaP. As for other endpoints, smaller NPs and MPs were more effective than 4.5 μm MPs in inducing AcP activity when compared at the same PS mass. On the other hand, exposure to BaP alone did not affect AcP activity in hemocytes thus indicating that AcP induction was mainly caused by PS particles and not by BaP. Sendra et al.^16^ also found increased tracking of lysosomes by flow cytometry in mussel granular hemocytes exposed for 3 h to PS particles of 0.1 and 1 μm in vitro. In an in vivo study, Revel et al.^66^ reported an enhancement of hemocytes AcP activity in mussels exposed to low and medium concentrations (0.008 and 10 μg/L) of a polydispersed mixture of PE MPs (0.4 to 720 μm) and PP MPs (0.4 to 950 μm). In another study in vivo*,* Avio et al.^31^ reported an up-regulation of different genes coding for lysosomal enzymes in mussels exposed to 50 μg/L of PE and PS MPs (100 to 1000 μm) alone and with pyrene. As mentioned before, the enhancement in AcP activity could be related to the increased endo-lysosomal activity upon exposure to PS NPs and MPs but it could also be due to an hypersynthesis of AcP and activation of cellular defenses as AcP is secreted as a humoral defense factor in mussel hemocytes^67^.

Exposure to PS particles alone did not induce intracellular ROS production in hemocytes at any concentration tested. Similar results were obtained in fish leucocytes exposed in vitro to PVC-PE MPs^68^. In murine immunocytes, Hwang et al.^57^ reported that PP MPs increased ROS production only at the highest concentration (1000 μg/mL). However, Wu et al.^54^ reported that PS MPs at 200 μg/mL significantly increased intracellular ROS production in the human Caco-2 cell line. In mussel hemocytes exposed to amino modified 50 nm PS NPs extracellular ROS levels increased significantly^18^ whereas a complex ROS response pattern was found by Sendra et al.^16^ for different subpopulations of mussel hemocytes and different PS plastic sizes. In our study, it could be that intracellular ROS production did not increase in hemocytes exposed to high concentrations of PS particles because these concentrations were cytotoxic to the cells. Another possible explanation could be that ROS production increased earlier than at 24 h, as shown by Sendra et al.^16^, and triggered antioxidant mechanisms which then reduced ROS levels at the 24 h time point. In support of this idea, in mussel hemocytes exposed in vitro to CdS quantum dots, intracellular ROS production significantly increased already at 1 h and then dropped up to 24 h^63^. In fact, the TEM finding that exposures to high concentrations of NPs and MPs caused an increase in residual bodies containing highly oxidized lipofuscin materials point to an oxidative stress situation as well as to enhanced lysosomal and autophagic activities^42^.

In cells exposed to PS NPs and MPs with BaP, ROS production increased at the highest concentrations tested for the three particle sizes. Exposure to BaP alone also enhanced ROS production in hemocytes at all three concentrations tested, in agreement with previous data in hemocytes in vitro^58,59^ and in mussels in vivo^69^. Intracellular ROS production is a consequence of the generation of reactive metabolites during BaP biotransformation, such as BaP diol epoxide, which is known to bind covalently to DNA, and BaP quinones which induce oxidative stress^70,71^. It has already been reported that exposure of bivalves in vivo to MPs with BaP may cause an increase in ROS production^23^ or in antioxidant catalase activity^22^. Therefore, our results suggest that the increase in ROS production in hemocytes exposed to high concentrations of PS NPs and MPs with BaP was mainly caused by BaP, confirming again that PS NPs and MPs acted as carriers of BaP to mussel hemocytes.

Partly due to ROS overproduction, BaP is a well-known genotoxic and carcinogenic agent and its ability to cause DNA strand breaks has been widely reported in bivalve species^69,72^. In agreement, in this study BaP alone was genotoxic at all concentrations tested, showing a dose-dependent response. Further, DNA damage significantly increased in cells treated with NPs and MPs alone or with BaP at the two concentrations tested for each PS particle size. Results showed higher levels of DNA damage in cells treated with NPs and MPs with BaP compared to cells treated with PS particles alone. Similar levels of DNA damage were observed in cells treated with BaP alone and in cells treated with NPs or MPs with BaP at equivalent BaP concentrations, indicating that BaP was the main inducer of genotoxicity. Recent studies have reported the capacity of MPs alone or with PAHs to induce genotoxicity in bivalves in vivo^22,66,73,74^. The mechanisms leading to genotoxicity of NPs and MPs are still not fully understood but seem to be related to increased ROS production and oxidative stress^73^. As discussed before, in this study NPs and MPs alone did not induce ROS production after 24 h exposure, however an earlier induction might have occurred, leading to development of genotoxic effects.

## Conclusions

Taken together our results indicate that PS particle size, ionic strength of the medium and presence of BaP were relevant factors influencing PS particle stability. Despite PS particles, and especially 0.05 μm NPs, largely aggregated in BME culture medium, individual particles of 0.05, 0.5 and 4.5 μm were identified within the cells exposed to the respective NPs or MPs. Cells appeared to internalize PS NPs and MPs alone or with BaP via different mechanisms depending on their size, and once in the cells, particles were found especially in large lysosomes containing lipofuscins, which were more abundant in cells exposed to PS particles, and also in the cytoplasm outside lysosomes. Results on the toxicity tests showed that PS particles alone or with BaP were cytotoxic (MTT assay) to hemocytes only at the highest concentrations tested. The same occurred for most sublethal endpoints determined except for increased phagocytic activity provoked by NPs and 0.5 μm MPs at lower concentrations. In general, at the same PS mass, smaller NPs and MPs exerted a higher toxicity compared to larger 4.5 μm MPs. Virgin PS NPs and MPs caused damage to the plasma membrane integrity, increased phagocytic and acid phosphatase activities and induced DNA damage. PS NPs and MPs with BaP decreased plasma membrane integrity and phagocytic activity, increased lysosomal AcP activity and induced intracellular ROS production and genotoxicity. Similar effects were observed in cells exposed to BaP alone. Overall, plastic particles appeared to be the main drivers for reduced plasma membrane integrity and increased phagocytic and lysosomal activities in hemocytes whereas BaP appeared to contribute more to reduced cell viability, inhibition of phagocytosis and induced ROS production and genotoxicity.

In this work we used PS NP and MP spheres as model materials to study their fate and toxicity in mussel cells but other environmentally relevant polymers and shapes need to be studied in the future for a comprehensive understanding of the risks posed by NP and MP pollution in the marine environment. For instance, PP and PE were the predominant polymers in bivalves and sediments in the Bay of Biscay (north-east of the Atlantic Ocean) and fragments were the most common MP type, followed by fibres (reviewed in Mendoza et al.^3^). Also weathering (aging) and biofilm formation in environmental plastics are important factors to take into consideration in future toxicological studies. Studies need to be conducted at environmentally realistic concentrations even though actual exposure levels and levels of MPs accumulated in biota are possibly underestimated^8^. As to the selected biological model, mussel hemocytes represent the most important front of immune defense in bivalves and also a suitable rapid screening tool for toxicity profiling and for mechanistic understanding of the mode of action of NPs and MPs and associated pollutants. Current conclusions drawn on the Trojan horse ability of NPs and MPs for BaP using this in vitro model agree well with in vivo studies^22,23,32^, pointing out to their potential use as predictive tools in environmental risk assessment. Finally, the fact that PS NPs and MPs can act as carriers of BaP to mussel cells and that presence of BaP enhanced the toxicity of PS particles, rise concerns about the risks posed by nano and microplastics in association with POPs to aquatic organisms.

## Methods

### Preparation of NPs and MPs with BaP

The plastic particles selected for the experiments were unlabelled polystyrene (PS) particles of three different sizes: nanoplastics (NPs) of 0.05 μm (Polysciences Inc., cat.# 08,691) and microplastics (MPs) of 0.5 and 4.5 μm (Polysciences Inc., cat.# 07,307 and cat.# 17,135, respectively). PS NPs and MPs were delivered at room temperature and stored at 4 °C.

MPs with sorbed BaP were prepared according to Batel et al.^51^ with modifications explained below. 0.5 µm MPs (10^9^ part./mL) and 4.5 µm MPs (10^8^ part./mL) were mixed with 1 µM BaP prepared in 0.001% DMSO in distilled water and left in agitation for 24 h in darkness in order to promote BaP sorption to the MPs. After that, suspensions were filtered (0.2 µm pore size) and retained MPs with sorbed BaP were recovered by passing cell culture media (Basal Medium Eagle (BME) supplemented with 1% gentamicin, 1040 mOsm/kg, pH 7.4) through the opposite side of syringe filters. As filtering and recovery of NPs was not possible due to their nanosize, 0.05 µm NPs (10^12^ part./mL) were directly mixed with 1 µM BaP prepared in 0.001% DMSO in supplemented BME and left in agitation for 24 h in darkness prior to the exposures. As previously reported by other authors^22,59,75^ DMSO at 0.001% is not cytotoxic to mussel cells. All suspensions were prepared in glass vials wrapped in aluminium foil.

### Characterization of NPs and MPs alone or with BaP in different media

Hydrodynamic size and surface charge of 0.05 μm NPs (10^12^ part./mL), 0.5 µm MPs (10^9^ part./mL) and 4.5 µm MPs (10^8^ part./mL) with or without BaP suspended in distilled water and in supplemented BME were assessed by DLS (Dynamic Light Scattering, Malvern Zetasizer Nano ZS, Malvern Instruments Ltd, Worcestershire, UK). 0.05 μm NPs with BaP were characterized only in BME because they were directly mixed with BaP and BME during the preparation procedure. Three measurements were done for each particle in each different media.

### Hemocytes primary culture and in vitro exposures

Mussels, *Mytilus galloprovincialis* (3.5–4.5 cm shell length) were collected in Plentzia, Bay of Biscay (43° 24′ 41.9′′ N 2° 57′ 01.1′′ W), a relatively clean site^76^. Animals were acclimatized for 2 days in aerated tanks with seawater (0.5 L/mussel at 18 °C), daily food supply (Marin Coraliquid Sera GmbH, Heinsberg, Germany) and 12 h photoperiod in the aquaria facility of the Cell Biology in Environmental Toxicology research group (CBET, UPV/EHU).

After acclimatization, hemolymph was withdrawn from mussel’s posterior adductor muscle under aseptic conditions in a vertical laminar airflow cabinet (Cultair BC100, Cultek S.L., Madrid, Spain). Hemolymph from 50 animals was pooled and diluted 9:1 in anti-aggregation solution (171 mM NaCl; 0.2 M Tris; 0.15% v/v HCl 1 N; 24 mM EDTA) to obtain 1 × 10^6^ cells/mL, > 95% viable according to trypan blue exclusion assay^76^. Cell suspensions were seeded into µ-Dish 35 mm glass-bottom petri dishes (Ibidi GmbH, Planegg, Germany) for confocal studies, into 96-well microplates for TEM studies or into glass-coated 96-well microplates for the toxicity assays. 96-well microplates were centrifuged (Beckman Coulter, Palo Alto, USA) at 270 *g* for 10 min at 4 °C in order to favour cells to attach. Cells were kept in culture media (Basal Medium Eagle 1040 mOsm/kg, pH 7.4, supplemented with 0.001% gentamicin) for 24 h at 18 °C in a Sanyo incubator (Osaka, Japan) to establish the primary cell cultures before performing the exposures.

For the intracellular localization studies, hemocytes were exposed for 24 h to 0.05 μm NPs (10^12^ part./mL), 0.5 μm MPs (10^9^ part./mL) and 4.5 μm MPs (10^8^ part./mL) alone or with BaP and to BaP (1 μM). For the toxicity assays, cells were exposed for 24 h to 0.05 μm NPs (10^2^, 10^3^, 10^5^, 10^6^, 10^8^, 10^9^ and 10^12^ part./mL), 0.5 μm MPs (10^2^, 10^3^, 10^5^, 10^6^, 10^8^ and 10^9^ part./mL) and 4.5 μm MPs (10^2^, 10^3^, 10^5^, 10^6^ and 10^8^ part./mL) alone or with BaP. These ranges of concentrations were selected to allow comparisons in terms of number of particles (10^2^ to 10^8^ part./mL of NPs or MPs) and in terms of mass of PS (10^9^ part./mL of 0.05 μm NPs = 10^6^ part./mL of 0.5 μm MPs = 10^3^ part./mL of 4.5 μm MPs correspond to 0.05 μg PS/mL and 10^12^ part./mL of 0.05 μm NPs = 10^9^ part./mL of 0.5 μm MPs = 10^6^ part./mL of 4.5 μm MPs correspond to 50 μg PS/mL). For the genotoxicity assessment (Comet Assay), cells were exposed for 24 h to 0.05 μm NPs (10^8^ and 10^12^ particles/mL), 0.5 μm MPs (10^8^ and 10^9^ part./mL) and 4.5 μm MPs (10^6^ and 10^8^ part./mL) alone or with BaP. In parallel, cells were exposed to BaP at 1 μM, 0.56 μM and 0.12 μM, which are the concentrations of BaP present in 10^12^ part./mL of 0.05 μm NPs with BaP and the concentrations of BaP adsorbed to 10^9^ part./mL of 0.5 μm MPs and 10^8^ part./mL of 4.5 μm MPs with adsorbed BaP respectively, according to Martínez-Álvarez et al.^35^. Cells kept in supplemented BME were used as negative controls.

### Intracellular fate of NPs and MPs alone or with BaP

Intracellular fate of NPs and MPs was assessed by transmission electron microscopy (TEM) and confocal fluorescence microscopy. For TEM analysis^77^, after exposures hemocytes were washed 3 times with PBS and detached from the microplates using trypsin/EDTA until cells rounded up, transferred to microtubes containing cell culture media and centrifuged at 270 *g* for 10 min at 4 °C. Afterwards, supernatants were removed and pellets were fixed in filtered seawater containing 2.5% glutaraldehyde at 4 °C for 1 h. Then, samples were postfixed with osmium tetroxide and ferrocyanide (1:1), cleaned in filtered seawater, dehydrated in an ethanol series and embedded in Epon. Ultrathin (70 nm) sections were cut in a Reichert-Jung Ultracut E ultramicrotome (Leica Microsystems; Wetzlar, Germany) and mounted on Cu grids coated with Formvar (Sigma Aldrich, St. Louis, USA). TEM imaging was performed on a high resolution JEOL 2000 microscope (JEOL Co., Tokyo, Japan) operated at 80 kV.

For confocal microscopy, after exposures cells were washed three times with PBS in order to eliminate PS particles and then hemocytes’ lysosomes were labelled with LysoTracker ™ Deep Red (ThermoFisher, Waltham, USA) according to manufacturer’s instructions. Cells were analyzed in brightfield and fluorescence emitted by the labelled lysosomes was detected at λ_excitation_ 647/ λ_emission_ 668 nm whereas fluorescence corresponding to the BaP aromatic rings was detected at λ_excitation_ 340/ λ_emission_ 488 nm ^51^. Images were merged and edited using the software ImageJ FIJI (National Institutes of Health, USA).

### In vitro toxicity assays

In vitro toxicity assays were carried out as described previously^63,77^. Cell viability was assessed using the MTT assay (Sigma-Aldrich, St. Louis, USA) and absorbance was read at 570 nm in a Biotek EL 312 microplate reader (Winooski, USA). Plasma membrane integrity was assessed using the 5-carboxyfluorescein diacetate acetoxymethyl ester (CFDA-AM) assay and fluorescence was measured at λ_excitation_ 485/ λ_emission_ 535 nm in a Bio-Tek FLx 800 microplate reader (Winooski, USA). Phagocytic activity was measured in the same absorbance microplate reader mentioned before at 550 nm. Lysosomal acid phosphatase (AcP) activity was quantified in the same absorbance microplate reader at 405 nm. Reactive oxygen species (ROS) production was detected using the Carboxy-H_2_DCFDA assay (ThermoFisher, Waltham, USA) and fluorescence was measured at λ_excitation_ 492–495/λ_emission_ 517–527 nm in the same fluorescence microplate reader mentioned before. Comet assay was performed as detailed before^63^ with the following modifications. Slides were stained with 1% GreenSafe Premium (NZYTech, Lisbon, Portugal) and observed under an Olympus BX61 fluorescence microscope (Olympus optical Co, Hamburg, Germany). 100 randomly selected cells were analyzed and scored using the OpenComet tool of the software ImageJ FIJI. Cells treated with hydrogen peroxide (100 µM) were used as positive controls for the ROS and Comet assays. All assays were repeated three times and in each case there were 6 replicates per experimental group.

Differences between control cells and cells exposed to different concentrations of the NP or MP treatments were analyzed through the Kruskal–Wallis test followed by the Dunn’s post-hoc test for multiple comparisons. Differences between treatments with or without BaP, at the same NP or MP concentration, were evaluated through the Mann–Whitney’s U test. Mann–Whitney’s U test was also used to compare responses obtained in cells treated with BaP with cells treated with NPs or MPs with BaP at the same BaP concentration. Significant differences were established at *p* < 0.05 in all cases. All statistical analyses were carried out using the SPSS statistical software (IBM SPSS Statistics 23, Chicago, USA).

## Supplementary Information

Supplementary Figure S1.

## Data Availability

The source data underlying Figures, Table and Supplementary Information are available from the authors upon request.
