# Supplementary material for: Polystyrene nanoplastics and microplastics can act as Trojan horse carriers of benzo(a)pyrene to mussel hemocytes in vitro
Source: Sci Rep. 2021 Nov 17;11:22396. doi: 10.1038/s41598-021-01938-4 (PMC8599475; doi:10.1038/s41598-021-01938-4)
Supplement: Supplementary file 1 — Supplementary Figure S1. [file 41598_2021_1938_MOESM1_ESM.docx]

**Supplementary Information**

**Figure S1.** Confocal fluorescence microscope images of hemocytes exposed to NPs and MPs alone or in combination with BaP and to BaP alone. Images show cells in brightfield, lysosomes labelled with LysoTracker ™ (red), BaP fluorescence (blue) and merged image of the three previous channels. Arrowheads indicate structures resembling aggregates of 0.5 μm MPs. Scale bars = 10 μm.
